# Xylanase VmXyl2 is involved in the pathogenicity of *Valsa mali* by regulating xylanase activity and inducing cell necrosis

**DOI:** 10.3389/fpls.2024.1342714

**Published:** 2024-04-29

**Authors:** Xinyue Cui, Xinke Li, Shen Li, Yan Huang, Na Liu, Sen Lian, Baohua Li, Caixia Wang

**Affiliations:** ^1^ Shandong Engineering Research Center for Environment-Friendly Agricultural Pest Management, College of Plant Health and Medicine, Qingdao Agricultural University, Qingdao, Shandong, China; ^2^ College of Horticulture, Qingdao Agricultural University, Qingdao, Shandong, China

**Keywords:** *Valsa mali*, xylanase VmXyl2, inducing cell necrosis, protein interaction, apple tree

## Abstract

Xylanase plays a key role in degrading plant cell wall during pathogenic fungi infection. Here, we identified a xylanase gene, *VmXyl2* from the transcriptome of *Valsa mali* and examined its function. *VmXyl2* has highly elevated transcript levels during the infection process of *V. mali*, with 15.02-fold increase. Deletion mutants of the gene were generated to investigate the necessity of *VmXyl2* in the development and pathogenicity of *V. mali*. The *VmXyl2* deletion mutant considerably reduced the virulence of *V. mali* in apple leaves and in twigs, accompanied by 41.22% decrease in xylanase activity. In addition, we found that VmXyl2 induces plant cell necrosis regardless of its xylanase activity, whereas promoting the infection of *V. mali* in apple tissues. The cell death-inducing activity of VmXyl2 dependent on BRI1-associated kinase-1 (BAK1) but not Suppressor of BIR1-1 (SOBIR1). Furthermore, VmXyl2 interacts with Mp2 *in vivo*, a receptor-like kinase with leucine-rich repeat. The results offer valuable insights into the roles of VmXyl2 in the pathogenicity of *V. mali* during its infection of apple trees.

## Introduction

Apple is a globally significant crop; however, the branch disease severely hampers the progress of the apple industry and leads to substantial economic losses for fruit farmers. *Valsa mali*, the pathogen responsible for apple tree canker, is a harmful fungus that primarily infects the branches and trunks of apple trees (Li et al., 2013). This pathogen mainly infects through the dead epidermal tissues and different types of wounds, particularly pruning wounds (Chen et al., 2016). The disease is characterized by the presence of abundant pycnidia on cankers, which can release conidia continuously throughout the year. *V. mali* is a representative necrotrophic fungus that can survive for extended periods on apple branches (Wang et al., 2018).

Necrotrophic fungi have the ability to infect living cells and tissues, as well as to grow and reproduce in host tissues. One of the primary mechanisms involves the secretion of enzymes and production of toxins to kill the host tissue and degrade its cell wall before invading, allowing fungi to subsequently enter the host (McCombe et al., 2022; Fei and Liu, 2023). Therefore, the pathogenic mechanism of *V. mali* is considered complex. The studies have shown that the cell wall-degrading enzymes (CWDEs) including xylanase, pectinase and β-glucosidase, as well as toxins likes protocatechuic acid and p-hydroxybenzoic acid, play an important role in the pathogenesis of *V. mali* (Wang et al., 2014; Yin et al., 2015; Xu et al., 2018; Yu et al., 2018).

Xylanase can destroy hemicellulose present in the plant cell walls. More specifically, it hydrolyzes xylan to xylose. Pathogens utilize xylose, which makes them conducive to infect the host (Yu et al., 2018). Xylanases are classified into several glycoside hydrolase (GH) families. According to the amino acid composition of the catalytic region of xylanase and the sequence of hydrophobic clusters, most of the known endonucleases belong to the GH10 and GH11 families (Pollet et al., 2010). Numerous studies have demonstrated xylanases play a pivotal role in the infection process of various pathogenic fungi. For example, in *Fusarium oxysporum* f. sp. *lycopersici*, the xylanase genes *Xyl3* and *Xyl4* persist throughout the infection process, whereas *Xyl5* is only expressed at the early stage of infection, and *Xyl2* is only expressed at the end of the infection (Ruiz-Roldán et al., 1999; Gómez-Gómez et al., 2001, 2002). The infectivity of *Mycosphaerella graminicola* has been demonstrated to have a strong correlation with its secretion of xylanase enzymes (Siah et al., 2007, 2009). Additionally, the disruption of the xylanase genes, *Xyn11A and BcXyl1*, in *Botrytis cinerea* resulted in a substantial decrease in its capacity to infect host plants (Brito et al., 2006). Subsequent studies have revealed that both Xyn11A and BcXyl1can induce plant cell death in several plants, regardless of their xylanolytic activities. However, Xyn11A promotes *B. cinerea* infection in tomatoes, while BcXyl1 confers resistance to *B. cinerea* in tomatoes (Noda et al., 2010; Yang et al., 2018).

Our previous research has revealed that the xylanase *VmXyl1*, encoding a xylanase which belongs to GH10 family, contributes to the pathogenicity of *V. mali* by specifically utilizing its xylanase activity (Yu et al., 2018). Transcriptome profiling revealed that *VmXyl2*, which encodes a xylanase belonging to GH11 family, was upregulated during *V. mali* infection, suggesting that this gene may play a significant role in fungal virulence. In this study, we generated gene deletion mutants and obatined purified protein of VmXyl2 to evaluate its involvement in the development and pathogenicity of *V. mali*. Moreover, we found that VmXyl2 induces plant cell necrosis, thereby facilitating *V. mali* infection in the host. Our findings suggest that *VmXyl2* plays a significant role in the virulence of *V. mali*, which provides valuable insights into the pathogenicity of necrotrophic fungi.

## Materials and methods

### Strains and culture conditions

The wild-type *V. mali* strain LXS080901 isolated and preserved by our laboratory was grown on potato dextrose agar (PDA) at 25°C in the dark. The gene deletion mutants and complementation strains were cultured on PDA supplemented with 100 mg/ml hygromycin B or geneticin G418 (Sigma, St. Louis, MO, USA). *Escherichia coli* strains were grown in Luria-Bertani (LB) with appropriate antibiotics at 37°C. *Agrobacterium tumefaciens* strains were grown in LB with appropriate antibiotics at 28°C.

### Identification and sequence analysis of *VmXyl2* in *V. mali*

Total RNA was isolated from fresh mycelia using RNAiso Plus Kit (TaKaRa, Dalian, China). Subsequently, 5 μg of total RNA from each sample was reverse transcribed to cDNA using a HiScript II 1st-Strand cDNA Synthesis Kit (+gDNA wiper) (Vazyme, Nanjing, China). The gene *VmXyl2*, which is predicted to have xylanase activity and shows high transcript levels during *V. mali* infection, was cloned and sequenced.

The amino acid sequences of xylanases from other strains in this study were obtained from the NCBI GenBank. All the homology searches were carried out on the NCBI BLAST server. The phylogenetic tree was inferred using the Maximum Likelihood (ML) method implemented in MEGA 7.0, with 1000 bootstrapping replicates. The DNAMAN 6.0 was used to perform multiple sequence alignments of VmXyl2 and other well-characterized xylanases from the GH11 family.

### Detection of gene expression by RT-qPCR

Mycelia grown on PDA for 3 days were used to inoculate apple twigs (Meng et al., 2021). The bark tissues were sampled at 0, 6, 12, 24, 48, 72, 96 and 120 hours post inocualtion (hpi). The RNA was extract from bark tissues, and then the cDNA was synthesized. All RT-qPCR experiments were conducted with SYBR Master Mix (TaKaRa, Dalian, China), following the manufacturer’s protocol. The *EF1-a* gene was used as an endogenous reference. The whole experiment was repeated twice, and three replicates were included in each experiment. All primers used in these assays are listed in **Supplementary Table S1**.

### Generation of gene deletion and complementation strains

To obtain *VmXyl2* gene deletion mutants, PEG-mediated protoplast transformation was carried out to get homologous recombination as described previously (Meng et al., 2021). The gene deletion cassette with three components used the hygromycin B phosphotransferase gene (*HPH*) as a selective marker for gene deletion (**Supplementary Figure S1**). Upstream and downstream fragments of VmXyl2 genes were amplified from genomic DNA of the wild-type strain LXS080901 using the gene-specific primers (**Supplementary Table S1**). The *HPH* gene was amplified from the vector pBS. The gene deletion cassette was generated by double-joint PCR, and the result was confirmed by sequencing. The cassettes were later transformed into the protoplasts of *V. mali* LXS080901, and the transformants were screened by culturing on medium with 100 μg/ml hygromycin B. The putative gene deletion mutants were validated by PCR using four primer pairs (**Supplementary Figure S1** and **Supplementary Table S1**). For generating the *VmXyl2* complementation strains, the fragment containing the full-length coding of *VmXyl2* and its native promoter region are constructed into pYF11 vector by homologous recombination. Then transformed into the *VmXyl2* deletion mutant through PEG-mediated transformation. The transformants were confirmed by PCR.

### Vegetative growth, pycnidia formation, and pathogenicity assays

Mycelial plugs (diameter of 5 mm) cut from actively growing colony edges of the wild-type strain, gene deletion mutants, and complemented mutants were transferred to PDA plates. The plates were then incubated at 25°C before the colony shape, color, and diameters were assessed. For the pycnidia formation experiment, the *V. mali* strains were cultured on PDA plates for 3 days at 25°C and then induced for 30 days under UV light (365 nm), and the number of pycnidia was counted.

Pathogenicity assays were performed using apple leaves and 1-year-old twigs (*M. domestica* ‘Fuji’) were collected from the greenhouse at Qingdao Agricultural University, Qingdao, China. The detached leaves and twigs were sterilized with 75% ethanol, and wounds were made as described by Yu et al. (2018). Mycelial plugs were used to inoculate the wounds. The inoculated leaves and twigs were placed in trays, which were maintained under conditions of high humidity, 25 °C and darkness. The lesion length was measured, and the development of the lesions was photographed at several time points. The assays were repeated three times, and at least 15 leaves and twigs were included in each treatment.

### Xylanase activity assay

The evaluation of xylanase activity was conducted following the 3,5-dinitrosalicylic acid (DNS) method described by Yu et al. (2018). This process involved a reaction mixture comprising the purified recombinant protein or culture filtrate and 0.5% beechwood xylan dissolved in 50 mM sodium citrate buffer at pH 5.0. The mixture was incubated at 50°C for 30 minutes. Subsequently, DNS solution was added to the mixture, which was then boiled for 5 minutes. Absorbance was measured at 540 nm. Xylanase activity was defined as the enzyme quantity necessary to catalyze the release of 1.0 mmol of xylose per minute under the conditions of pH 5.0 and 50°C. The activity was quantified in units per minute per milligram of protein (U/mg).

### Recombinant protein expression and purification

Full length VmXyl2 cDNA was amplified and cloned into was amplified and cloned and inserted into the *Ecor*I and *BssH*II sites of the pET-32a vector. VmXyl2 recombinant protein was expressed in *E. coli* strain DE3 cells. Expression was induced by incubation with 0.3 mM isopropyl-β-D-thiogalactopyranoside (IPTG) for 24 h at 16°C. Cells were collected by centrifugation at 5000 × g for 10 min. For protein extraction, cells were resuspended in lysis buffer (20 mM sodium hydrogen phosphate, 300 mM NaCl, pH 7.4) supplemented with 1 mg/ml lysozyme, 1 mM phenylmethanesulfonyl fluoride (PMSF), and 1.98 mM β-mercaptoethanol and then subjected to sonication and centrifugation at 10,000 × g for 10 min. VmXyl2 was purified by affinity chromatography using Ni-NTA resin (Thermo Scientific, Waltham, MA, USA) following the manufacturer’s instructions.

### Plant growth and agro-infiltration

Tobacco plants were grown in a climate chamber (16-h photoperiod, 22°C, 65% relative humidity). *A. tumefaciens* strain GV3101 carrying pGR106 or TRV vectors cultured as above. The bacterial cells were pelleted and resuspended in MES buffer (10 mM MgCl_2_, 1.0 mM MES, and 200 μ acetosyringone, pH 5.7) in the dark for 3 h at room temperature before infiltration. For agro-infiltration assays, *A. tumefaciens* cells in suspension were combined with a silencing suppressor at a suitable ratio to achieve a final optical density (OD_600_) of 0.6. Subsequently, this mixture was infiltrated into the leaves of 6-week-old tobacco plants using a syringe without its needle attached.

For Tobacco Rattle Virus (TRV)-mediated gene silencing, *A. tumefaciens* cultures expressing TRV2 constructs and those expressing TRV1 were mixed at a 1:1 ratio to a final OD_600_ of 0.8 and then infiltration into primary leaves of four-leaf-stage tobacco seedlings. BCL2-associated X (BAX) and empty vector were used as controls. Three weeks after treatment with TRV2 constructs, plants were used for corresponding assays.

### Yeast two-hybrid assay

Y2H assays for examining the interactions among pGAD-MP1/MP2 and pGBK-VmXyl2. pGADT7 and pGBKT7 fused with specific genes were cointroduced into the yeast competent cell AH109 strains (Weidi, Shanghai, China). Yeast cotransformants expressing the bait and prey constructs were isolated on SD-Leu-Trp plates for 2 days and screened by culturing on SD-Trp-Leu-His-Ade plates with 1 mM X-α-gal for 3 days. Yeast transformants co-transformed with pGADT7‐T and pGBKT7‐53 were used as positive controls, and transformants co-transformed with pGADT7‐T and pGBKT7‐Lam served as negative controls.

### Bioinformatics analysis

The molecular weight of VmXyl2 protein were analyzed and predicted using https://prosite.expasy.org/prosite.html. Signal peptides were analyzed with DetaiBio (http://www.detaibio.com/tools/index.php?r=signal-peptide/index). The hydrophobic amino acid sequence was analyzed with https://web.expasy.org/protparam/. The relationships among the xylanase protein family were analyzed through a Blast analysis of the NCBI website (https://blast.ncbi.nlm.nih.gov/Blast.cgi).

### Statistical analysis

All treatments were performed in three independent biological experiments with three replicates. All statistical analysis was conducted using SPSS software (Version 19.0, SPSS Inc., Shanghai, China). All the data collected were subjected to analysis of variance (ANOVA) followed by Duncan’s multiple range tests. The asterisks indicate a statistically significant difference with the wild-type strain (*p < 0.05; **p < 0.01).

## Results

### Identification and expression profile of *VmXyl2* in *V. mali*

The gene *VmXyl2* was amplified by PCR using cDNA of *V. mali* as a template and were confirmed by sequencing. The cDNA of *VmXyl2* contains an open reading frame of 681 bp that encodes a protein with 226 amino acid and a calculated molecular mass of 23.84 kDa. Using the Signal P5.0 server, VmXyl2 was predicted that the first 19 amino acids are its signal peptides.

**Figure 1:**
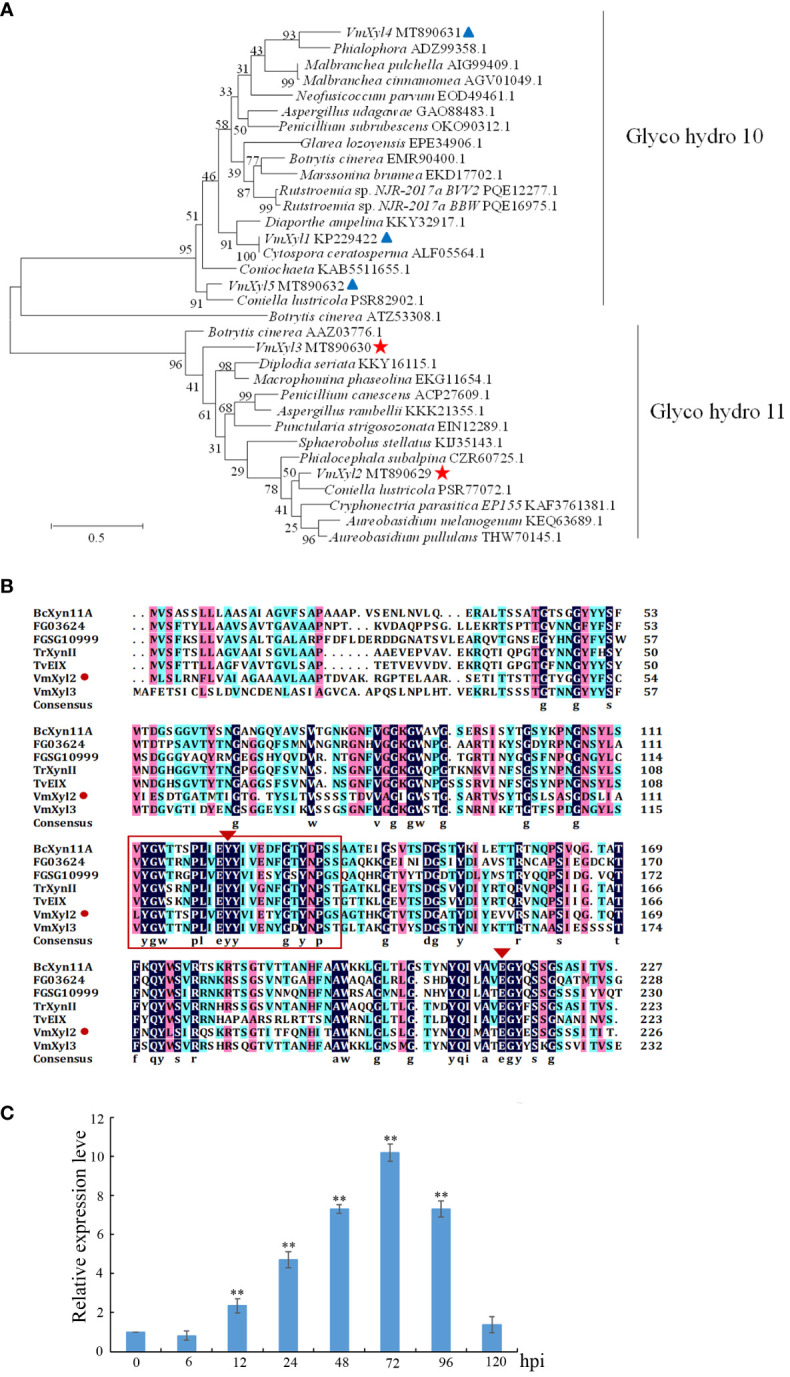
Phylogenetic tree and alignment of the amino acid sequences of xylanases. **(A)** Phylogenetic analysis of xylanases belonging to GH10 and GH11 families. The phylogram was generated using the maximum-likelihood algorithm as implemented in MEGA 7. The numbers beside each node indicate bootstrap values as a percentage of 1,000 bootstraps. Species names are followed by the accession numbers of xylanase protein. **(B)** Amino acid sequence alignment of VmXyl2 with five known xylanases from GH11 family. The aligned sequences were obtained from the phylogenetic tree. The orange rectangles indicate conserved motifs of xylanases. **(C)** Transcript levels of *VmXyl2* at different time points post-inoculation determined by quantitative PCR (qPCR). The transcript level of *V. mali EF1-a* was used as an internal control, and the transcript level of *VmXyl2* in the mycelia grown on potato dextrose agar (PDA) was standardized to 1. The means and standard deviation of the relative expression levels were calculated from three independent biological replicates. Relative expression levels of *VmXyl2* at 0, 6, 12, 24, 48, 72, and 120 hpi. Asterisks represent significant differences (**p < 0.01) in transcript levels as compared to that at 0 hpi.

Phylogenetic tree was constructed with the characterized xylanase proteins from various strains. The genomic sequence of *V. mali*, as published, revealed the presence of five candidate genes that are responsible for encoding xylanases (Yin et al., 2015). Phylogenetic analysis indicated that five genes encoding xylanase in *V. mali* belonged to two families (**Figure 1A**). VmXyl1, VmXyl4, and VmXyl5 are members of the GH10 family, while VmXyl2 and VmXyl3 are members of GH11 family. The sequence alignment of VmXyl2 and other xylanase from GH11 family showed that VmXyl2 possesses the catalytic residues associated with their activity. Specifically, Glu122 and Glu213 serve as the catalytic sites for enzyme activity (**Figure 1B**). Both VmXyl2 and the reference sequences exhibit a conserved motif consisting of 25 amino acids.

The transcript levels of *VmXyl2* at various time points (0, 6, 12, 24, 48, 72, 96, and 120 hpi) were determined during infection of apple twigs by *V. mali* (**Figure 1C**). The transcript level of *VmXyl2* was significantly enhanced from 12 hpi, gradually increased and reached the highest with a 15.02-fold change at 72 hpi. Overall, the significant upregulation of *VmXyl2* during infection indicates its potential involvement in the pathogenicity of *V. mali*.

### *VmXyl2* is not required for vegetative growth but pycnidia formation of *V. mali*

To conduct the functional analysis of *VmXyl2* in *V. mali*, we generated a mutant strain with a targeted gene deletion (**Supplementary Figure S1A**). These transformants were further confirmed by PCR using the primer pairs presented in **SupplementaryTable S1** (**Supplementary Figure S1B**). Corresponding results obtained from these assays showed that *VmXyl2* was successfully replaced with *HPH.* In addition, the complementation strain *ΔVmXyl2-C* was created by introducing the native promoter of the *VmXyl2* coding region into the *ΔVmXyl2* mutant. The complementation strain *ΔVmXyl2-C* was verified by PCR (**Supplementary Figure S1C**).

**Figure 2:**
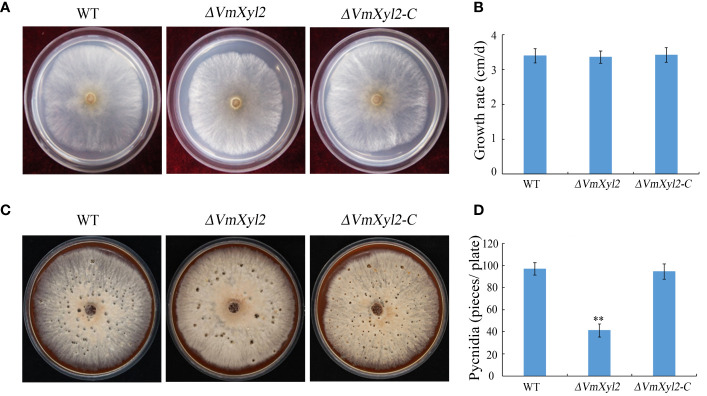
Colony morphology, vegetative growth and pycnidia formation of wild-type strain, *VmXyl2* deletion strain, and complementation strain. **(A)** Colony phenotypes of different strains grown on PDA at 25°C in the dark for 2 days. **(B)** Mycelial growth rate of different strains on PDA at 25°C for 2 days. **(C)** Colony phenotypes of different strains grown on bark culture medium at 25°C for 15 days under UV light (365 nm). **(D)** Pycnidia number of different strains produced on per plate under UV light. The bars represent the standard deviations, and the asterisks indicate significant differences (**p < 0.01) in the gene deletion mutant compared with the wild-type strain.

To investigate the influence of *VmXyl2* on the growth and development of *V. mali*, we evaluated the colony morphology, growth rate, and pycnidia formation of the gene deletion mutant *ΔVmXyl2* and the wild-type strain. However, we did not observe any significant differences in colony morphology or growth rate between *ΔVmXyl2* and the wild-type strain (**Figures 2A, B**). All strains were able to form pycnidia on PDA under UV-light (365 nm). The *ΔVmXyl2* strain produced fewer than 41 pycnidia per plate, whereas the wild-type strain produced over 96 pycnidia per plate. Additionally, the complementation strain *ΔVmXyl2-C* by reintroducing *VmXyl2* restored pycnidia formation to that of the wild-type strain (**Figures 2C, D**). The results suggested that *VmXyl2* has no impact on the vegetative growth, but it does affect the pycnidia formation of *V. mali*.

### Deletion of *VmXyl2* reduces the pathogenicity of *V. mali*

**Figure 3:**
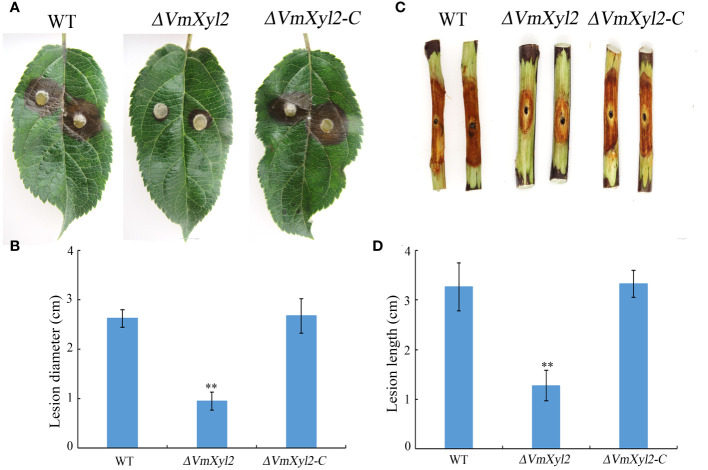
Pathogenicity assays of wild-type strain, *VmXyl2* deletion strain, and complementation strain in apple leaves and twigs. **(A, C)** The infected phenotype in apple leaves and twigs inoculated with different strains. **(B, D)** Lesion sizes cauased by different strains in apple leaves and twigs. Asterisks on bars indicate a significant difference with the wild-type strain (**p < 0.01). The experiments were repeated thrice.

Pathogenicity assays were conducted on the detached apple leaves and twigs to investigate the role of *VmXyl2* in disease development. The results indicated that the *ΔVmXyl2* strain exhibited significantly reduced virulence towards apple leaves and twigs, in contrast to the wild-type strain (**Figure 3**). The wild-type strain typically exhibited symptoms of necrosis and canker. The *ΔVmXyl2* strain exhibited a reduction of over 63.81% and 60.98% in the average lesion size on both apple leaves and twigs. Moreover, the complementation strain *ΔVmXyl2-C* restored the highly virulent phenotype, exhibiting the same symptoms on both apple leaves and twigs. The results indicated that *VmXyl2* plays a crucial role in the pathogenicity of *V. mali*.

### Deletion of *VmXyl2* reduced xylanase activity in *V. mali*

**Figure 4:**
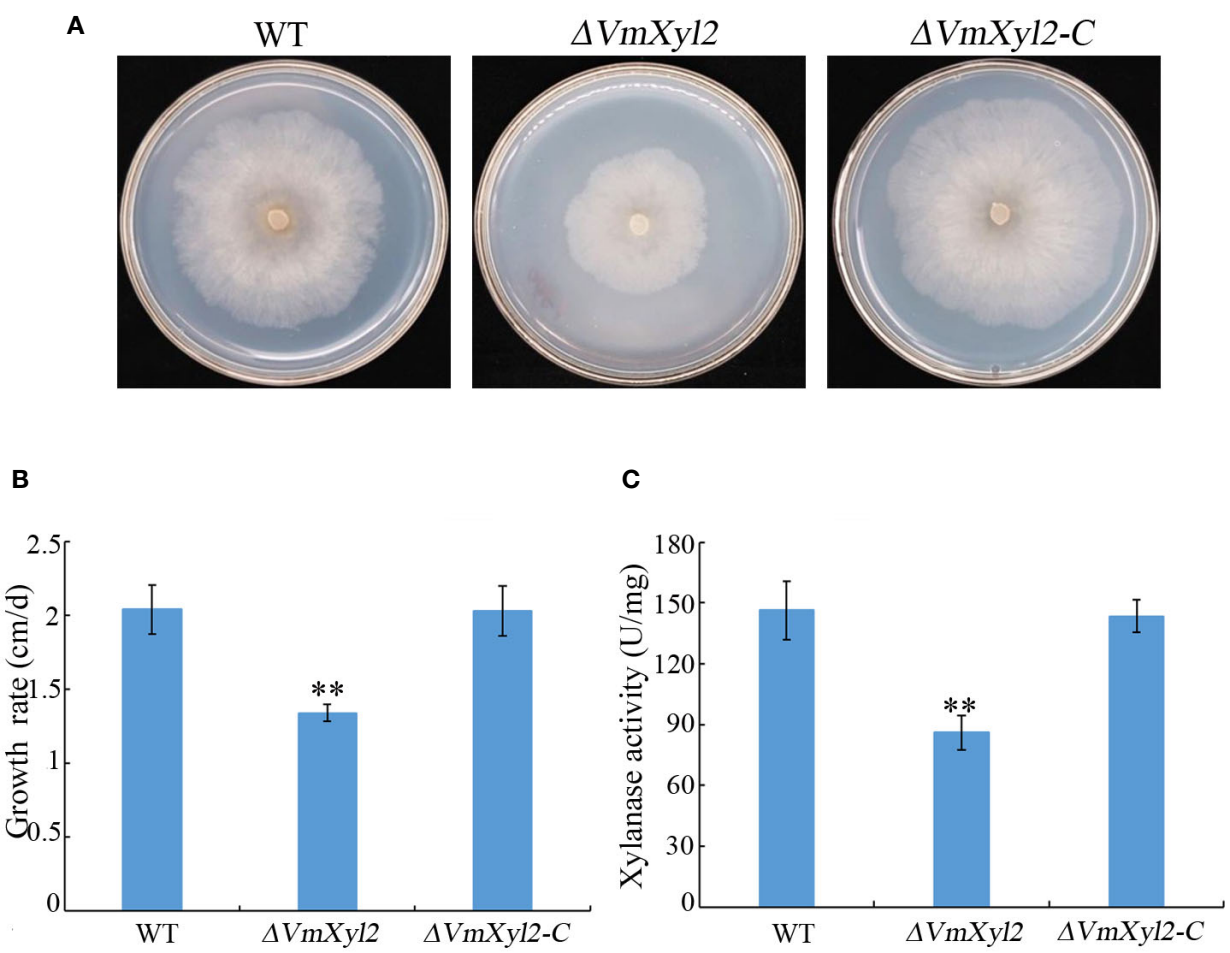
Xylan utilization and xylanase activity assays of wild-type strain, *VmXyl2* deletion strain, and complementation strain. **(A)** Colony phenotypes of different strains grown on minimal medium supplemented with xylan as a sole carbon source for 5 days at 25°C. **(B)** Mycelial growth rate of different strains on minimal medium supplemented with xylan as a sole carbon source. **(C)** Xylanase activities in the culture filtrates of wild-type strain, *VmXyl2* deletion strain and complementation strain. The xylanase activity was expressed as units per min per mg protein (U/mg). The bars represent the standard deviations, and the asterisks indicate significant differences with the wild-type strain (**p < 0.01).

To assess the effect of *VmXyl2* deletion on the ability of *V. mali* to utilize xylan, we compared the growth rate of the wild-type and mutant strains on minimal medium supplemented with xylan as a sole carbon source. The growth rate of *ΔVmXyl2* mutant was significantly affected by the deletion of *VmXyl2*, the deletion mutant grew 34.31% slower than the wild-type strain (**Figures 4A, B**). We further examined the effect of deleting *VmXyl2* on xylanase activity in the culture filtrates of the wild-type and mutant strains. Xylan was found to induce the production and secretion of xylanase. The xylanase activity of the *ΔVmXyl2* mutant was reduced 41.22% compared to the wild-type stain (**Figure 4C**). The reintroduction of the native gene *VmXyl2* resulted in the restoration of growth rate and xylanase activity to the levels comparable to the wild-type strain (**Figure 4**). The results indicated that VmXyl2 is involved in the absorption of xylan and carry xylanase activity in *V. mali*.

### VmXyl2 induces plant cells necrosis

**Figure 5:**
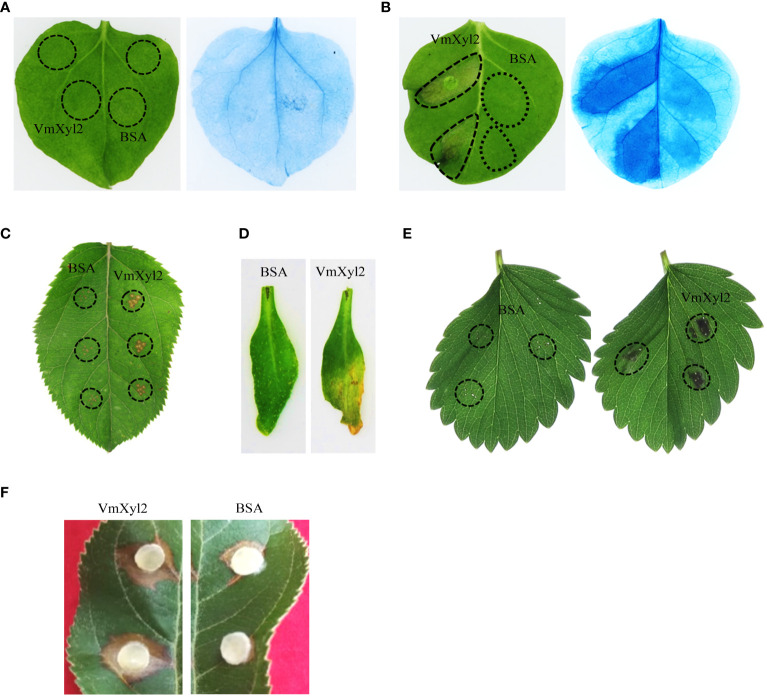
VmXyl2 inducing cell necrosis in several plants. **(A)** *N. benthamiana* leaves infiltrated with purified VmXyl2 and detected by trypan blue staining. **(B)** *N. tabacum* cv. Samsun leaves infiltrated with purified VmXyl2 and detected by trypan blue staining. **(C)** *M. domestica* leaves treated with purified VmXyl2. **(D)** *A. thaliana* leaves treated with purified VmXyl2. **(E)** *F. ananassa* leaves treated with purified VmXyl2. **(F)** *M. domestica* leaves treated with purified VmXyl2 and inoculated with *V. mali* wild-type strain. BSA was used as control.

The recombinant protein VmXyl2 was successfully purified to study its ability to induce cell death in both host and non-host plants (**Supplementary Figure S2A**). VmXyl2 was found to cause significant cell necrosis in the leaves of *Nicotiana tabacum* cv. Samsun, *M. domestica*, *Arabidopsis thaliana* and *Fragaria ananassa*, while no necrotic effects were observed in *N. benthamiana* leaves (**Figures 5A–E**). Cell necrosis was observed at a minimum concentration of 50 nM VmXyl2. The size of necrosis increased with higher concentrations of purified protein, ranging from 50 nM to 200 nM (**Supplementary Figure S2B**). Moreover, VmXyl2 was discovered to stimulate the accumulation of hydrogen peroxide (**Supplementary Figure S2C**) and the upregulation of defense-related genes in tobacco and apple leaves (**Supplementary Figure S3**).

To investigate the impact of VmXyl2-inducing cell necrosis on *V. mali* infection, the pathogen was inoculated to apple leaves following treatment with purified protein. Bovine serum albumin (BSA) was used as a control at the same concentration to VmXyl2. Compared to BSA treatment, the lesion size exhibited a significant increase after VmXyl2 treatment (**Figure 5F**), which indicates that VmXyl2-inducing cell necrosis promotes the infection of *V. mali* on apple leaves.

### The signal peptide and conserved region C^111-135^ are crucial for cell death-inducing activity of VmXyl2

**Figure 6:**
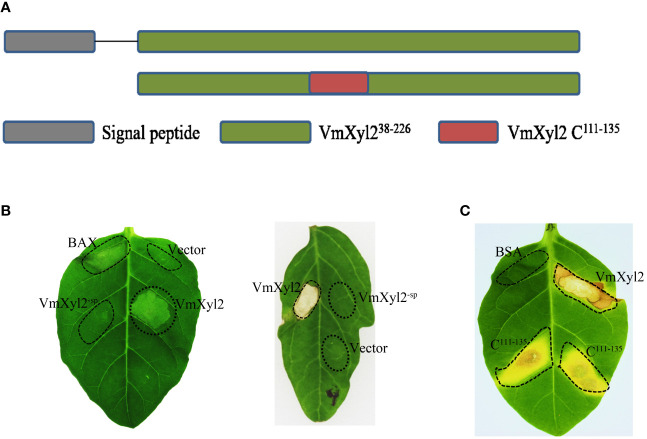
Effects of the signal peptide and conserved region on VmXyl2 function. **(A)** Schematic presentation of the examined constructs, including signal peptide and conserved region. **(B)** Analysis of cell necrosis in *N. tabacum* cv. Samsun and tomato leaves by transiently expressing the full length VmXyl2 and VmXyl2-^SP^ (without signal peptide). **(C)** Analysis of cell necrosis in *N. tabacum* cv. Samsun infiltrated with a 25-residue pepide (C^111-135^).

VmXyl2 contains a signal peptide encoding 19 amino acids at N-terminus, implying VmXyl2 might be a secreted protein (**Figure 6A**). In order to confirm the previous hypothesis that VmXyl2 is a secreted protein to induce cell death, we transiently expressed the full length VmXyl2 and VmXyl2^-SP^ (without signal peptide) in tobacco and tomato plants through agro-infiltration. The result showed that VmXyl2, which contains signal peptide, was able to induce cell necrosis in *N. tabacum* cv. Samsun and tomato leaves. However, the absence of the signal peptide in VmXyl2^-sp^ eliminated its abiltiy to induce cell death (**Figure 6B**). The results suggest that the signal peptide is essential for VmXyl2-mediated cell death.

Plant receptors frequently recognize specific small protein epitopes to induce plant cell death. The amino acid sequence analysis revealed that the conserved sequence of VmXyl2, which consists of a 25-residue pepide (C^111-135^), shares 81.71% identity with five other xylanases (**Figure 1B**). To elucidate the function of the 25-residue peptide, we synthetically produced this small peptide segment. It was found that the conserved region C^111-135^ induced the same cell death as full-length VmXyl2 in *N. tabacum* cv. Samsun leaves (**Figure 6C**).

The sequence alignment revealed that VmXyl2 contains two potentially highly conserved catalytic residues (E122 and E213), which are crucial for its xylanase activity (**Figure 4A**). To investigate whether the ability of VmXyl2 to induce plant cell necrosis is dependent on its hydrolase activity, we obtained the site-directed mutant protein that two glutamic acid residues were substituted by aspartate (**Supplementary Figure S4B**). Enzymatic assays with site-directed mutant protein showed that xylanase activity was nearly eliminated (**Supplementary Table S2**). Surprisingly, the mutant protein lacked xylanase activity but still retained the cell death-inducing activity, similar to the wild-type protein (VmXyl2). The mutant protein and VmXyl2 caused similar visible cell death symptoms in *N. tabacum* cv. Samsun and tomato leaves (**Supplementary Figures S4C, D**). The results suggests that the cell death-inducing activity of VmXyl2 is independent of its hydrolase activity.

### BAK1 but not SOBIR1 is required for VmXyl2-inducing cell necrosis

**Figure 7:**
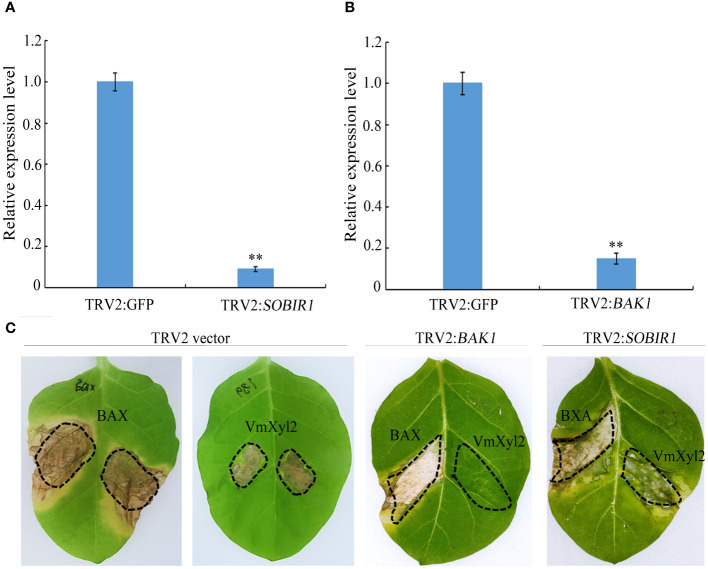
BAK1 not SOBIR1 mediated VmXyl2-inducing cell death in *N. tabacum* cv. Samsun. **(A, B)** Silencing efficiency of *SOBIR1* and *BAK1* in *N. tabacum* cv. Samsun leaves examined by qPCR.The bars represent the standard deviations, and the asterisks indicate significant differences (**p < 0.01) at the expression level. **(C)** Transient expression of VmXyl2 in *BAK1* and *SOBIR1* silenced *N. tabacum* cv. Samsun leveas, respectively. BAX was used as the positive control.

BRI1-associated kinase-1 (BAK1) and Suppressor of BIR1-1 (SOBIR1) are the Leucine-Rich Repeats Receptor-Like Kinase (LRR-RLK), and it was found that BcXyl1-inducing cell death was mediated by BAK1 and SORBIR1 (Yang et al., 2018). To explore the mechanism of VmXyl2-inducing cell necrosis, we silenced the expression levels of *BAK1* and *SOBIR1* in tabacco plants. The qPCR analysis validated a significant reduction in the expression levels of *BAK1* or *SOBIR1* upon inoculation with TRV::*BAK1* or TRV::*SOBIR1*, showing an expression level less than 20% compared to inoculation with TRV::GFP (**Figures 7A, B**). After three weeks of viral inoculation to silence *BAK1* in *N. tabacum* cv. Samsun, the transient expression of VmXyl2 through agro-infiltration with VmXyl2 expression constructs did not induce cell necrosis. However, the results obtained from plant with silenced *SOBIR1* demonstrated that VmXyl2 induced cell death, which was inconsistent with the findings in plants with silenced *BAK1* (**Figure 7C**). In all assays, the positive control BAX induced cell death in plants. Based on these results, we concluded that BAK1, but not SOBIR1, was necessary for VmXyl2-inducing cell death in *N. tabacum* cv. Samsun.

### VmXyl2 interacts with Mp2

**Figure 8:**
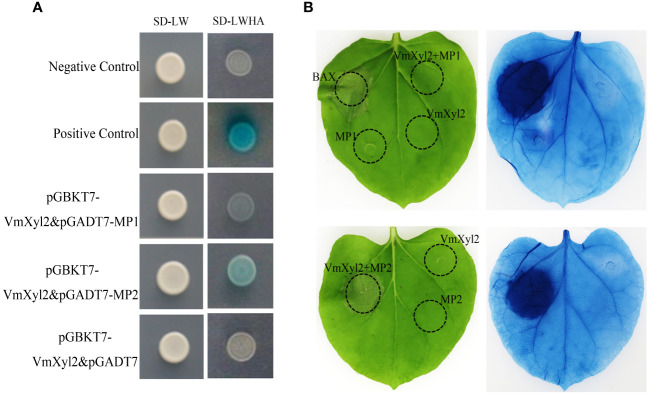
Identification of the interaction of VmXyl2 and MP2. **(A)** The interactions between pGADT7-MP1 or pGADT7-MP2 and pGBKT7-VmXyl2 exmained by yeast two-hybrid assay. Yeast cotransformants expressing the bait and prey constructs were isolated on SD-Leu-Trp plates for 2 days and screened by culturing on SD-Trp-Leu-His-Ade plates with 1 mM X-α-gal for 3 days. Yeast transformants cotransformed with pGADT7‐T and pGBKT7‐53 were used as positive controls, and transformants cotransformed with pGADT7‐T and pGBKT7‐Lam served as negative controls. **(B)** The interaction between MP1 or MP2 and VmXyl2 confirmed through infiltration assays. VmXyl2 and Mp1 or Mp2 were co-infiltrated into *N. benthamiana* leaves and stained with trypan blue for visualization. BAX was as the positive control.

The tomato LRR-RLK, LeEix2, can recognize xylanase TvEIX and induce cell death in plants (Ron and Avni, 2004; Pizarro et al., 2018b). By analyzing the whole-genome sequence of *M. domestica*, we obtained two candidate LRR-RLKs, named Mp1 and Mp2, which exhibited the highest homology to LeEix2. The yeast two-hybrid method was used to verify the interaction between two LRR-RLKs and VmXyl2. The results showed that only Mp2 exhibited interaction with VmXyl2 *in vitro* (**Figure 8A**). To further confirm the interaction *in vivo*, we performed a tobacco infiltration experiment. Previous experiments showed that VmXyl2 alone did not induce cell necrosis in *N. benthamiana* leaves (**Figure 5A**). We subsequently co-infiltrated VmXyl2 with Mp1 or Mp2 into the leaves of *N. benthamiana*. The results showed that cell necrosis was observed only in leaves co-infiltrated with VmXyl2 and Mp2 (**Figure 8B**). This suggests an *in vivo* interaction between VmXyl2 and Mp2, and highlights the requirement of Mp2 for VmXyl2-induced cell necrosis in *N. benthamiana* leaves.

## Discussion

CWDEs are an essential group of enzymes secreted by pathogenic fungi, which play crucial roles in the procession of pathogen invasion, establishment, and reproduction (Rafiei et al., 2021). Xylanases, as one of the primary CWDEs, have been demonstrated to participate in pathogenicity, specifically in the infection and development of necrotrophic fungi (Brito et al., 2006; Kubicek et al., 2014). The objective of the present study was to investigate the crucial virulence CWDEs in apple Valsa canker pathogen (*V. mali*), with a specific focus on xylanases, which are widely distributed among various fungal species (Brito et al., 2006; Yu et al., 2016). The genomic sequence that has been published predicts the presence of five candidate genes encoding xylanases (Yin et al., 2015). The transcription analysis revealed that all five genes were upregulated to varying degrees, with *VmXyl1* and *VmXyl2* exhibiting the most significant increase (**Supplementary Figure S5**). In the previous study, we investigated the function of *VmXyl1* and found that its involvement in the pathogenicity of *V. mali* (Yu et al., 2018). In this study, we characterized *VmXyl2* and elucidated its involvement in *V. mali* infection.

Increasing studies have reported that xylanases are crucial pathogenicity factors in the pathogenesis of necrotrophic fungi, including *V. mali* (Yu et al., 2018). For instance, when the genes encoding xylanases in *Sclerotinia sclerotiorum* and *B. cinerea* were knocked out, the mutant strains showed decreased xylanase activities, which in turn affected their ability to infect plants (Brito et al., 2006; Yu et al., 2016; Yang et al., 2018). This study highlights the importance of *VmXyl2* as a key pathogenicity factor in *V. mali*. The significant upregulation of this gene during pathogen infection, as well as the substantial reduction in pathogenicity observed in the mutant with this gene deleted, provide compelling evidence for its functional role.

In this study, we observed that *VmXyl2* deletion did not affect morphology and mycelial growth rate, but it was found to be essential for pycnidia formation in *V. mali*. Similar results have been demonstrated in other pathogenicity factors of pathogenic fungi (Xu et al., 2018). For example, the deletion mutant of *VmXyl1*, which is another gene encoding xylanase, displayed a normal growth rate and reduction in pycnidia formation (Yu et al., 2018). Moreover, the deletion of *BcKMO*, which encodes kynurenine 3-monooyxgenase, in *B. cinerea* is known to play a crucial role in fungal development. The mutant lacking *BcKMO* exhibits impaired conidia and sclerotia production (Zhang et al., 2018). Conidia production is a vital phase in the pathogens’ life cycle (Huang et al., 2021). Therefore, it is widely believed that inhibiting pycnidia formation of *V. mali* could alleviate or effectively control the occurrence of Valsa canker in apple trees.

A significant finding of this work is that VmXyl2 exhibits both hydrolytic activity and the capacity to induce necrosis in plant cells. Interestingly, the induction of cell death does not depend on its enzymatic activity. This is consistent with the previous results of Xyn11A and BcXyl1 in *B. cinerea*, the cell death inducing activities were found to be unrelated to their enzymatic activities (Brito et al., 2006; Yang et al., 2018). In contrast, the enzymatic activity of cutinase VdCUT11 in *Verticillium dahliae* was necessary for inducing cell death (Gui et al., 2017). In addition, VmXyl2 induces cell necrosis in various plants, including both host and non-host species, except for *N. benthamiana*. One potential explanation could be the lack of a recognition receptor in *N. benthamiana* capable of interacting with VmXyl2, or it could be due to poor interaction with the receptor protein (Ron and Avni, 2004).

Generally, small peptides located on the protein surface can effectively stimulate an immune response, resulting in cell necrosis. For instance, the key structural domain of VdEG3 in *V. dahliae*, containing a GH12 domain, is capable of inducing cell death in *N. benthamiana* leaves (Gui et al., 2017). Similar results were also observed in *B. cinerea*, specailly a 25-residue peptide from BcXyn11A, induced cell death in tobacco leaves (Frías et al., 2019) In this study, we found that a 25-residue pepide (C^111-135^) from VmXyl2 exhibits a significant similarity to BcXyn11A. Moreover, this peptide effectively induces cell necrosis in plants, further validating the conserved function of the GH11 xylanases.

Research on biotrophic and hemibiotrophic pathogens has shown that secretory protein or effectors can induce cell death, thereby enhancing plant resistance and limiting pathogens invasion (Xiang et al., 2021). However, necrotrophic fungi, which require the disruption of host cells or tissues prior to infection, can exploit cell necrosis to promote their infection and invasion (Hofius et al., 2007; Oliver and Solomon, 2010). A previous study demonstrated that xylanase Xyn11A from *B. cinerea* enhances virulence by inducing cell necrosis in the plant tissue surrounding the infection (Noda et al., 2010). Similarly, the secretory protein Vd424y from another necrotrophic fungus *V. dahliae* induced BAK1- and SOBIR1-dependent cell death and activated both salicylic acid and jasmonic acid signalling (Liu et al., 2021). In this study, the results revealed that VmXyl2 induced cell necrosis in apple leaves and promoted the expansion of lesions caused by *V. mali*. These findings suggest that *V. mali*, being a necrotrophic fungus, may exploit necrotic plant cells to facilitate its invasion process.

Previous studies have shown that RLKs, such as BAK1, and receptor-like proteins (RLPs), such as SOBIR1, are involved in the recognition of various pathogens (Yeh et al., 2016; van der Burgh et al., 2019; Wei et al., 2022). Such as, BcXyl1, VdCUT11, and XEG1 inducing cell death in plants was mediated by the plant BAK1 and SOBIR1 (Ma et al., 2015; Gui et al., 2017; Yang et al., 2018). The results in this study for VmXyl2 demonstrated that BAK1 but not SOBIR1 is the essential factor for its ability to induce cell death. Additionally, research finding has shown that the tomato LRR-RLK LeEix2 recognizes xylanases TvEIX, leading to the induction of programmed cell death (Ron and Avni, 2004; Leibman-Markus et al., 2017; Pizarro et al., 2018a). Based on the results, we hypothesize that LRR-RLKs in *M. domestica* can potentially recognize VmXyl2 because of its high identity with xylanase TvEIX. In order to confirm the hypotheis, we selected two apple LRR-RLKs that exhibit high homology to LeEix2, and validated the interation between xylanase VmXyl2 and apple MP2. However, the recognition and interaction mechanism of MP2 and VmXyl2 deserves to be further investigated.

## Data availability statement

The datasets presented in this study can be found in online repositories. The names of the repository/repositories and accession number(s) can be found in the article/**Supplementary Material**.

## Author contributions

XC: Writing – original draft, Writing – review & editing. XL: Writing – original draft, Writing – review & editing. ShL: Writing – original draft, Writing – review & editing. YH: Writing – original draft. NL: Writing – review & editing. SeL: Writing – review & editing. BL: Writing – review & editing. CW: Writing – review & editing.

## Supplementary material

Supplementary Figure 1 The generation of *VmXyl2* knockout mutants and complementation strains in *V. mali*. **(A)** Schematic diagram of *VmXyl2* targeted disruption, depicted in proportion. The black square represents the coding region, the other squares represent homologous regions adjacent to the constructed fragment of the hygromycin resistance gene *HPH*. **(B)** Gene deletion verification of *VmXyl2* by PCR using gene specific primers. Lanes 1 and 2 represent wild-type strain, lanes 3 to 6 represent putative transformants with *VmXyl2* deletion. Lanes 1, 3, and 5 were confirmed using primers of upstream fragments and target genes, lanes 2, 4, and 6 were confirmed using primers of downstream fragments and target genes. **(C)** Gene deletion verification of *VmXyl2* by PCR using *HPH* primers Lane 1 represents wild-type strain, lanes 2 and 3 represent putative transformants with *VmXyl2* deletion. **(D)** Detection of complementation strain by PCR. All the primers used are listed in **Supplementary Table S1**.

Supplementary Figure 2 VmXyl2 inducing cell necrosis and H_2_O_2_ accumulation. **(A)** Protein purification of VmXyl2 expressed in *E. coli*. **(B)** *N. tabacum* cv. Samsun leaves infiltrated with different concentrations of purified VmXyl2 and stained by trypan blue. **(C)** H_2_O_2_ accumulation detected by DAB staining in *N. tabacum* cv. Samsun leaves infiltrated with purified or transiently expressing VmXyl2.

Supplementary Figure 3 The relative expression levels of *NbAcre31*, *NbPti5*, *MdPR1*, and *MdPR5* examined by qPCR. The standard deviations are represented by the bars, and significant differences (** *p* < 0.01) in transcript levels are indicated by asterisks.

Supplementary Figure 4 The effect of xylanase activity on the cell death-inducing activity of VmXyl2. **(A)** Schematic diagram of xylanase activity site in VmXyl2. **(B)** Prokaryotic expression of site-directed mutant proteins. M represents protein standard, Lane 1 represents uninduced pET-32a, Lane 2 represents uninduced of pET-*VmXyl2*, Lane 3 represents E122D site-directed mutant protein of VmXyl2; Lane 4 represents E213D site-directed mutant protein of VmXyl2; Lane 5 represents E122D and E213D double site-directed mutant protein of VmXyl2. **(C)** Tomato leaves infiltration with site-directed mutant protein and wild-type protein. **(D)** *N. tabacum* cv. Samsun leaves infiltrated with site-directed mutant protein and or transiently expressing protein.

Supplementary Figure 5 Transcript levels of *VmXyl2* at 72 h post inoculation of *V. mali* in apple twigs by qPCR. The transcript level of *V. mali EF1-a* was used as an internal control, and the transcript level of *VmXyl2* in the mycelia grown on PDA was standardized to 1. The means and standard deviation of the relative expression levels were calculated from three independent biological replicates. Different letters on bars indicate a statistically significant difference (** *p* < 0.01).

## References

[B1] Brito N. Espino J. J. González C. (2006). The endo-beta-1,4-xylanase xyn11A is required for virulence in Botrytis cinerea. Mol. Plant Microbe Interact. 19, 25–32. doi: 10.1094/MPMI-19-0025 16404950

[B2] Chen C. Li B. Dong X. Wang C. Lian S. Liang W. (2016). Effects of temperature, humidity, and wound age on *Valsa Mali* infection of apple shoot pruning wounds. Plant Dis. 100, 2394–2401. doi: 10.1094/PDIS-05-16-0625-RE 30686168

[B3] Fei W. Liu Y. (2023). Biotrophic fungal pathogens: a critical overview. Appl. Biochem. Biotechnol. 195, 1–16. doi: 10.1007/s12010-022-04087-0 35951248

[B4] Frías M. González M. González C. Brito N. (2019). A 25-residue peptide from *Botrytis cinerea* xylanase BcXyn11A elicits plant defenses. Front. Plant Sci. 10. doi: 10.3389/fpls.2019.00474 PMC6477079 31057580

[B5] Gómez-Gómez E. Isabel M. Roncero G. Di Pietro A. Hera C. (2001). Molecular characterization of a novel endo-beta-1,4-xylanase gene from the vascular wilt fungus. Fusarium oxysporum. Curr. Genet. 40, 268–275. doi: 10.1007/s00294-001-0260-0 11795847

[B6] Gómez-Gómez E. Ruíz-Roldán M. C. Di Pietro A. Roncero M. I. Hera C. (2002). Role in pathogenesis of two endo-beta-1,4-xylanase genes from the vascular wilt fungus *Fusarium oxysporum* . Fungal Genet. Biol. 35, 213–222. doi: 10.1006/fgbi.2001.1318 11929211

[B7] Gui Y. Chen J. Zhang D. Li N. Li T. Zhang W.. (2017). *Verticillium dahliae* manipulates plant immunity by glycoside hydrolase 12 proteins in conjunction with carbohydrate-binding module 1. Environ. Microbiol. 19, 1914–1932. doi: 10.1111/1462-2920.13695 28205292

[B8] Hofius D. Tsitsigiannis D. I. Jones J. D. Mundy J. (2007). Inducible cell death in plant immunity. Semin. Cancer Biol. 17, 166–187. doi: 10.1016/j.semcancer.2006.12.001 17218111

[B9] Huang Y. Yu C. Sun C. Saleem M. Li P. Li B.. (2021). β-Glucosidase VmGlu2 contributes to the virulence of *Valsa Mali* in apple tree. Front. Microbiol. 12. doi: 10.3389/fmicb.2021.695112 PMC8361449 34394036

[B10] Kubicek C. P. Starr T. L. Glass N. L. (2014). Plant cell wall-degrading enzymes and their secretion in plant-pathogenic fungi. Annu. Rev. Phytopathol. 52, 427–451. doi: 10.1146/annurev-phyto-102313-045831 25001456

[B11] Leibman-Markus M. Schuster S. Avni A. (2017). LeEIX2 interactors’ analysis and EIX-mediated responses measurement. Methods Mol. Biol. 1578, 167–172. doi: 10.1007/978-1-4939-6859-6_13 28220423

[B12] Liu L. Wang Z. Li J. Wang Y. Yuan J. Zhan J.. (2021). *Verticillium dahliae* secreted protein Vd424Y is required for full virulence, targets the nucleus of plant cells, and induces cell death. Mol. Plant Pathol. 22, 1109–1120. doi: 10.1111/mpp.13100 34233072 PMC8358993

[B13] Ma Z. Song T. Zhu L. Ye W. Wang Y. Shao Y.. (2015). A *Phytophthora sojae* glycoside hydrolase 12 protein is a major virulence factor during soybean infection and is recognized as a PAMP. Plant Cell 27, 2057–2072. doi: 10.1105/tpc.15.00390 26163574 PMC4531360

[B14] McCombe C. L. Greenwood J. R. Solomon P. S. Williams S. J. (2022). Molecular plant immunity against biotrophic, hemibiotrophic, and necrotrophic fungi. Essays Biochem. 66, 581–593. doi: 10.1042/EBC20210073 35587147 PMC9528087

[B15] Meng L. Sun C. Gao L. Saleem M. Li B. Wang C. (2021). Hydroxybenzoate hydroxylase genes underlying protocatechuic acid production in *Valsa Mali* are required for full pathogenicity in apple trees. Mol. Plant Pathol. 22, 1370–1382. doi: 10.1111/mpp.13119 34390112 PMC8518569

[B16] Noda J. Brito N. González C. (2010). The *Botrytis cinerea* xylanase Xyn11A contributes to virulence with its necrotizing activity, not with its catalytic activity. BMC Plant Biol. 10, 38. doi: 10.1186/1471-2229-10-38 20184750 PMC2844071

[B17] Oliver R. P. Solomon P. S. (2010). New developments in pathogenicity and virulence of necrotrophs. Curr. Opin. Plant Biol. 13, 4. doi: 10.1016/j.pbi.2010.05.003 20684067

[B18] Pizarro L. Leibman-Markus M. Schuster S. Bar M. Avni A. (2018a). SlPRA1A/RAB attenuate EIX immune responses via degradation of LeEIX2 pattern recognition receptor. Plant Signal Behav. 13, e1467689. doi: 10.1080/15592324.2018.1467689 29944445 PMC6103275

[B19] Pizarro L. Leibman-Markus M. Schuster S. Bar M. Meltz T. Avni A. (2018b). Tomato prenylated RAB acceptor protein 1 modulates trafficking and degradation of the pattern recognition receptor LeEIX2, affecting the innate immune response. Front. Plant Sci. 9. doi: 10.3389/fpls.2018.00257 PMC5838007 29545816

[B20] Pollet A. Delcour J. A. Courtin C. M. (2010). Structural determinants of the substrate specificities of xylanases from different glycoside hydrolase families. Crit. Rev. Biotechnol. 30, 176–191. doi: 10.3109/07388551003645599 20225927

[B21] Rafiei V. Vélëz H. Tzelepis G. (2021). The role of glycoside hydrolases in phytopathogenic fungi and oomycetes virulence. Int. J. Mol. Sci. 22, 9359. doi: 10.3390/ijms22179359 34502268 PMC8431085

[B22] Ron M. Avni A. (2004). The receptor for the fungal elicitor ethylene-inducing xylanase is a member of a resistance-like gene family in tomato. Plant Cell 16, 1604–1615. doi: 10.1105/tpc.022475 15155877 PMC490049

[B23] Ruiz-Roldán M. C. Di Pietro A. Huertas-González M. D. Roncero M. I. (1999). Two xylanase genes of the vascular wilt pathogen *Fusarium oxysporum* are differentially expressed during infection of tomato plants. Mol. Gen. Genet. 261, 530–536. doi: 10.1007/s004380050997 10323234

[B24] Siah Deweer C. Duyme F. Sanssené J. Durand R. Reignault P.. (2009). In planta xylanase activity and pathogenicity on wheat-*Mycosphaerella graminicola* pathosystem. Commun. Agric. Appl. Biol. Sci. 74, 693–700. 20222552

[B25] Siah A. Deweer C. Reignault P. Halama P. (2007). Two *Mycosphaerella graminicola* French isolates differ in symptoms, in planta sporulation and cell wall degrading enzymes *in vitro* production. Commun. Agric. Appl. Biol. Sci. 72, 867–874. 18396822

[B26] van der Burgh A. M. Postma J. Robatzek S. Joosten M. (2019). Kinase activity of SOBIR1 and BAK1 is required for immune signalling. Mol. Plant Pathol. 20, 410–422. doi: 10.1111/mpp.12767 30407725 PMC6637861

[B27] Wang C. Li C. Li B. Li G. Dong X. Wang G.. (2014). Toxins produced by *Valsa Mali* var. Mali and their relationship with pathogenicity. Toxins (Basel) 6, 1139–1154. doi: 10.3390/toxins6031139 24662481 PMC3968381

[B28] Wang X. Pan T. Lian S. Wang C. Li B. (2018). Effects of environmental factors on the growth and extension of *Valsa Mali* in the xylem of apple branches. China Agri Sci. 51, 3291–3301. doi: 10.3864/j.issn.0578-1752.2018.17.005

[B29] Wei X. Wang Y. Zhang S. Gu T. Steinmetz G. Yu H.. (2022). Structural analysis of receptor-like kinase SOBIR1 reveals mechanisms that regulate its phosphorylation-dependent activation. Plant Commun. 3, 100301. doi: 10.1016/j.xplc.2022.100301 35529948 PMC9073325

[B30] Xiang G. Yin X. Niu W. Chen T. Liu R. Shang B.. (2021). Characterization of CRN-like genes from *Plasmopara viticola*: Searching for the most virulent ones. Front. Microbiol. 12. doi: 10.3389/fmicb.2021.632047 PMC8044898 33868192

[B31] Xu M. Gao X. Chen J. Yin Z. Feng H. Huang L. (2018). The feruloyl esterase genes are required for full pathogenicity of the apple tree canker pathogen *Valsa Mali* . Mol. Plant Pathol. 19, 1353–1363. doi: 10.1111/mpp.12619 28960871 PMC6638109

[B32] Yang Y. Yang X. Dong Y. Qiu D. (2018). The *Botrytis cinerea* xylanase BcXyl1 modulates plant immunity. Front. Microbiol. 9. doi: 10.3389/fmicb.2018.02535 PMC6206051 30405585

[B33] Yeh Y. H. Panzeri D. Kadota Y. Huang Y. C. Huang P. Y. Tao C. N.. (2016). The *Arabidopsis* Malectin-like/LRR-RLK IOS1 is critical for BAK1-dependent and BAK1-independent pattern-triggered immunity. Plant Cell 28, 1701–1721. doi: 10.1105/tpc.16.00313 27317676 PMC5077175

[B34] Yin Z. Liu H. Li Z. Ke X. Dou D. Gao X.. (2015). Genome sequence of valsa canker pathogens uncovers a potential adaptation of colonization of woody bark. New Phytol. 208, 1202–1216. doi: 10.1111/nph.13544 26137988

[B35] Yu C. Li T. Shi X. Saleem M. Li B. Liang W.. (2018). Deletion of endo-β-1,4-xylanase VmXyl1 impacts the virulence of *Valsa Mali* in apple tree. Front. Plant Sci. 9. doi: 10.3389/fpls.2018.00663 PMC5966579 29868105

[B36] Yu Y. Xiao J. Du J. Yang Y. Bi C. Qing L. (2016). Disruption of the gene encoding endo-β-1, 4-xylanase affects the growth and virulence of *Sclerotinia sclerotiorum* . Front. Microbiol. 7. doi: 10.3389/fmicb.2016.01787 PMC5103160 27891117

[B37] Zhang K. Yuan X. Zang J. Wang M. Zhao F. Li P.. (2018). The kynurenine 3-monooxygenase encoding gene, *BcKMO*, is involved in the growth, development, and pathogenicity of *Botrytis cinerea* . Front. Microbiol. 9. doi: 10.3389/fmicb.2018.01039 PMC5968091 29867912

