# Supplementary figures and images for: Xylanase VmXyl2 is involved in the pathogenicity of Valsa mali by regulating xylanase activity and inducing cell necrosis
Source: Front Plant Sci. 2024 Apr 29;15:1342714. doi: 10.3389/fpls.2024.1342714 (PMC11092374; doi:10.3389/fpls.2024.1342714)

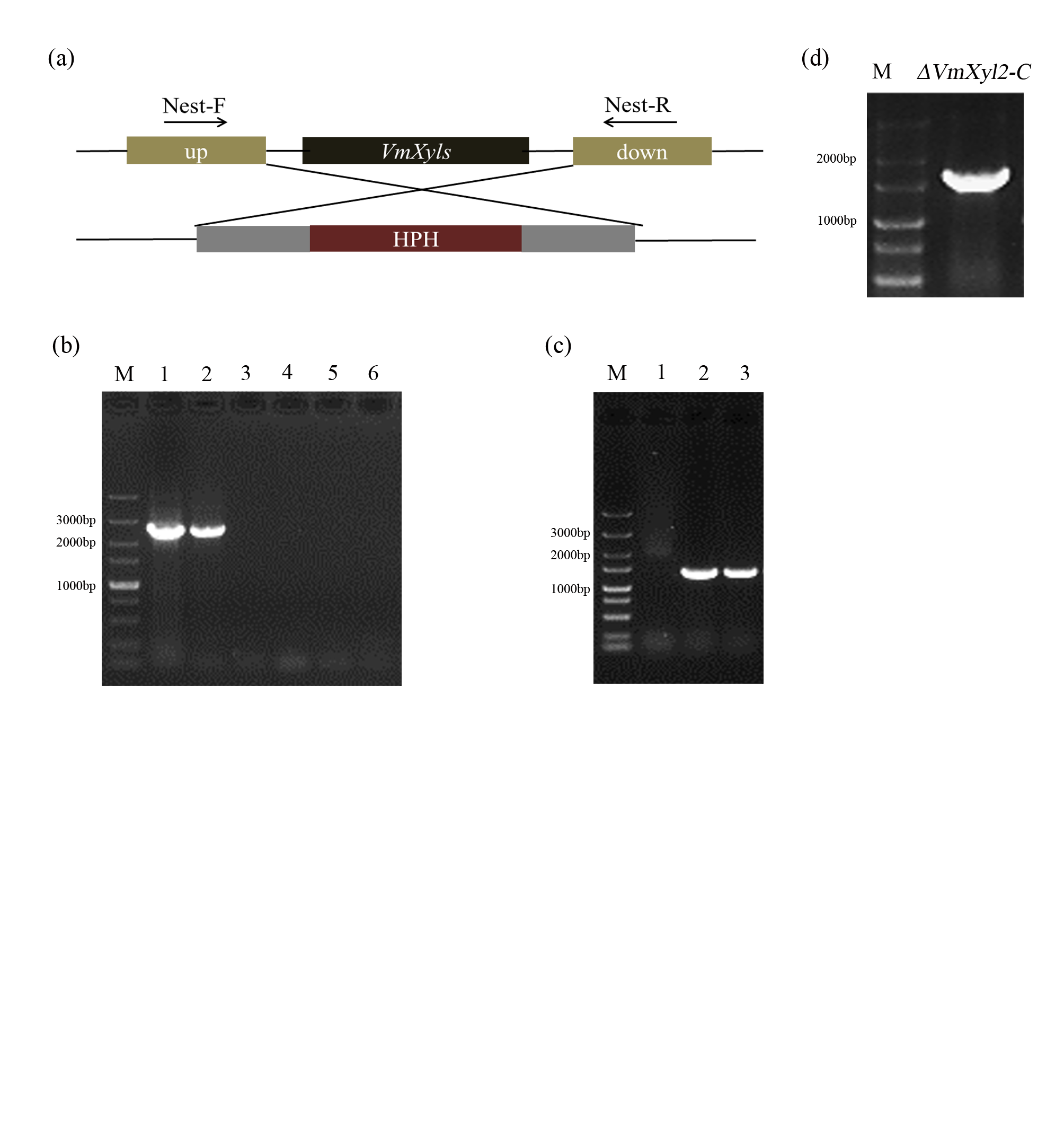

Supplement: Supplementary Figure 1 — The generation of VmXyl2 knockout mutants and complementation strains in V. mali. (A) Schematic diagram of VmXyl2 targeted disruption, depicted in proportion. The black square represents the coding region, the other squares represent homologous regions adjacent to the constructed fragment of the hygromycin resistance gene HPH. (B) Gene deletion verification of VmXyl2 by PCR using gene specific primers. Lanes 1 and 2 represent wild-type strain, lanes 3 to 6 represent putative transformants with VmXyl2 deletion. Lanes 1, 3, and 5 were confirmed using primers of upstream fragments and target genes, lanes 2, 4, and 6 were confirmed using primers of downstream fragments and target genes. (C) Gene deletion verification of VmXyl2 by PCR using HPH primers Lane 1 represents wild-type strain, lanes 2 and 3 represent putative transformants with VmXyl2 deletion. (D) Detection of complementation strain by PCR. All the primers used are listed in Supplementary Table S1 . [file Image_1.tif]

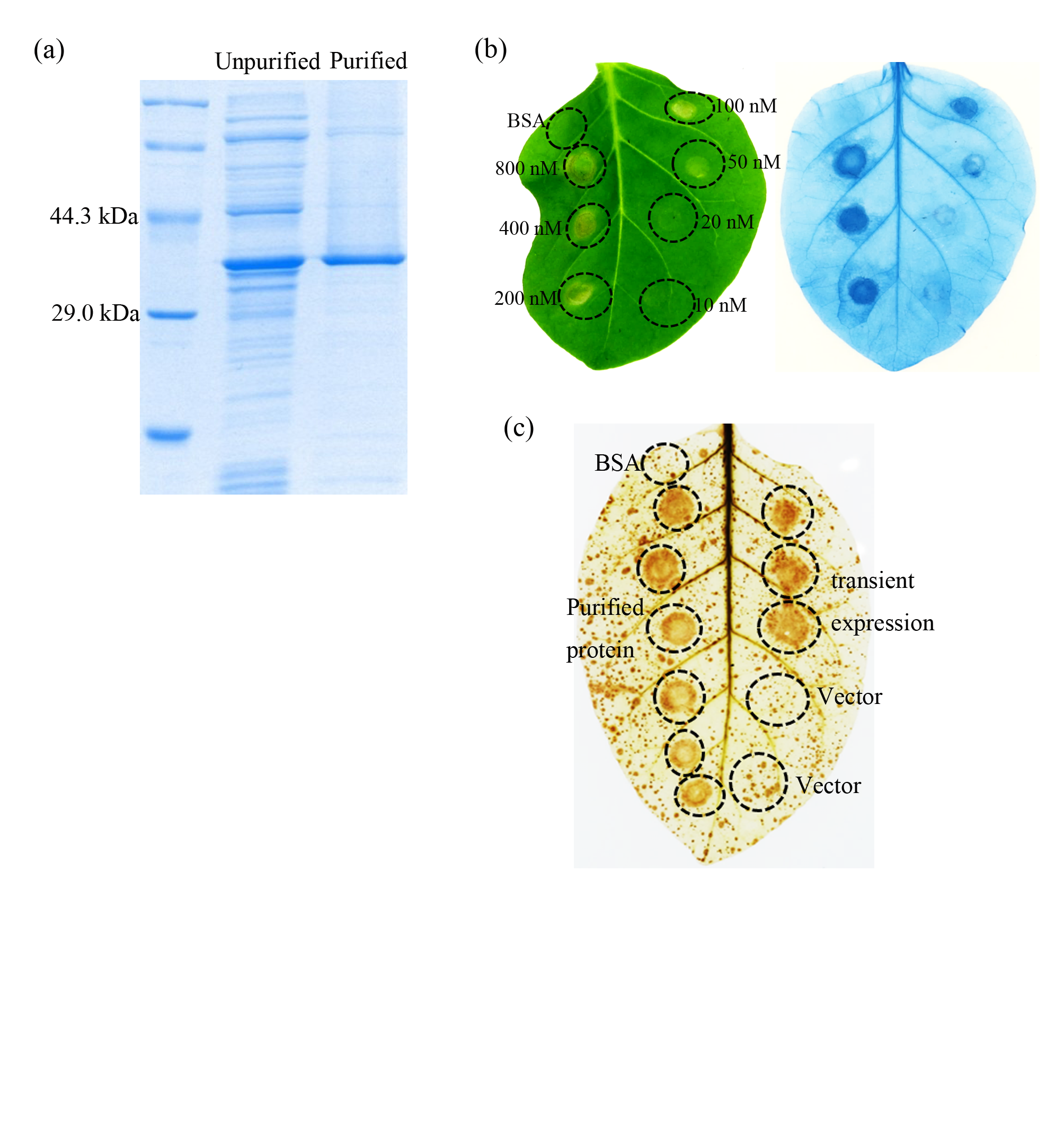

Supplement: Supplementary Figure 2 — VmXyl2 inducing cell necrosis and H2O2 accumulation. (A) Protein purification of VmXyl2 expressed in E. coli. (B) N. tabacum cv. Samsun leaves infiltrated with different concentrations of purified VmXyl2 and stained by trypan blue. (C) H2O2 accumulation detected by DAB staining in N. tabacum cv. Samsun leaves infiltrated with purified or transiently expressing VmXyl2. [file Image_2.tif]

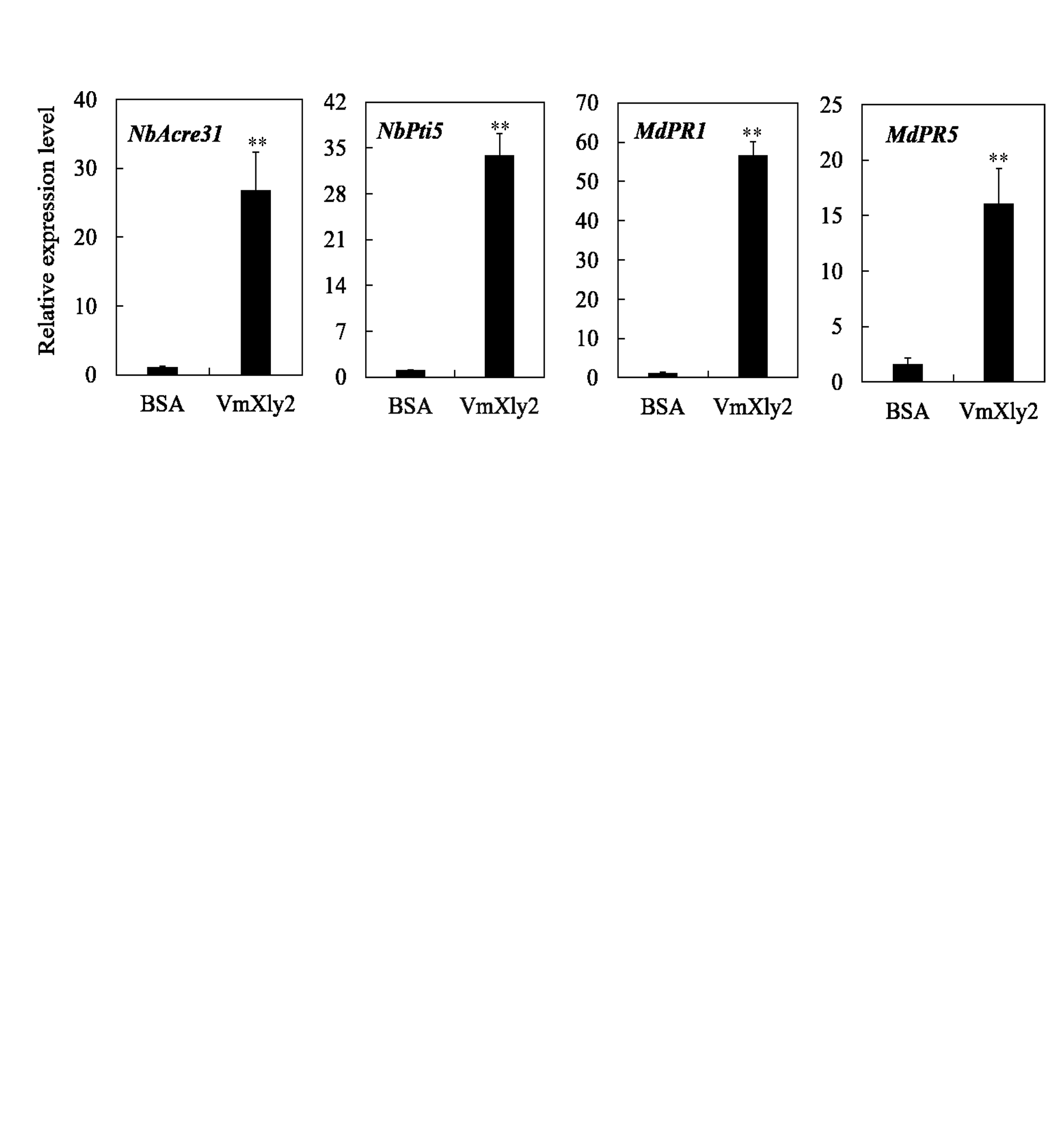

Supplement: Supplementary Figure 3 — The relative expression levels of NbAcre31, NbPti5, MdPR1, and MdPR5 examined by qPCR. The standard deviations are represented by the bars, and significant differences (** p < 0.01) in transcript levels are indicated by asterisks. [file Image_3.tif]

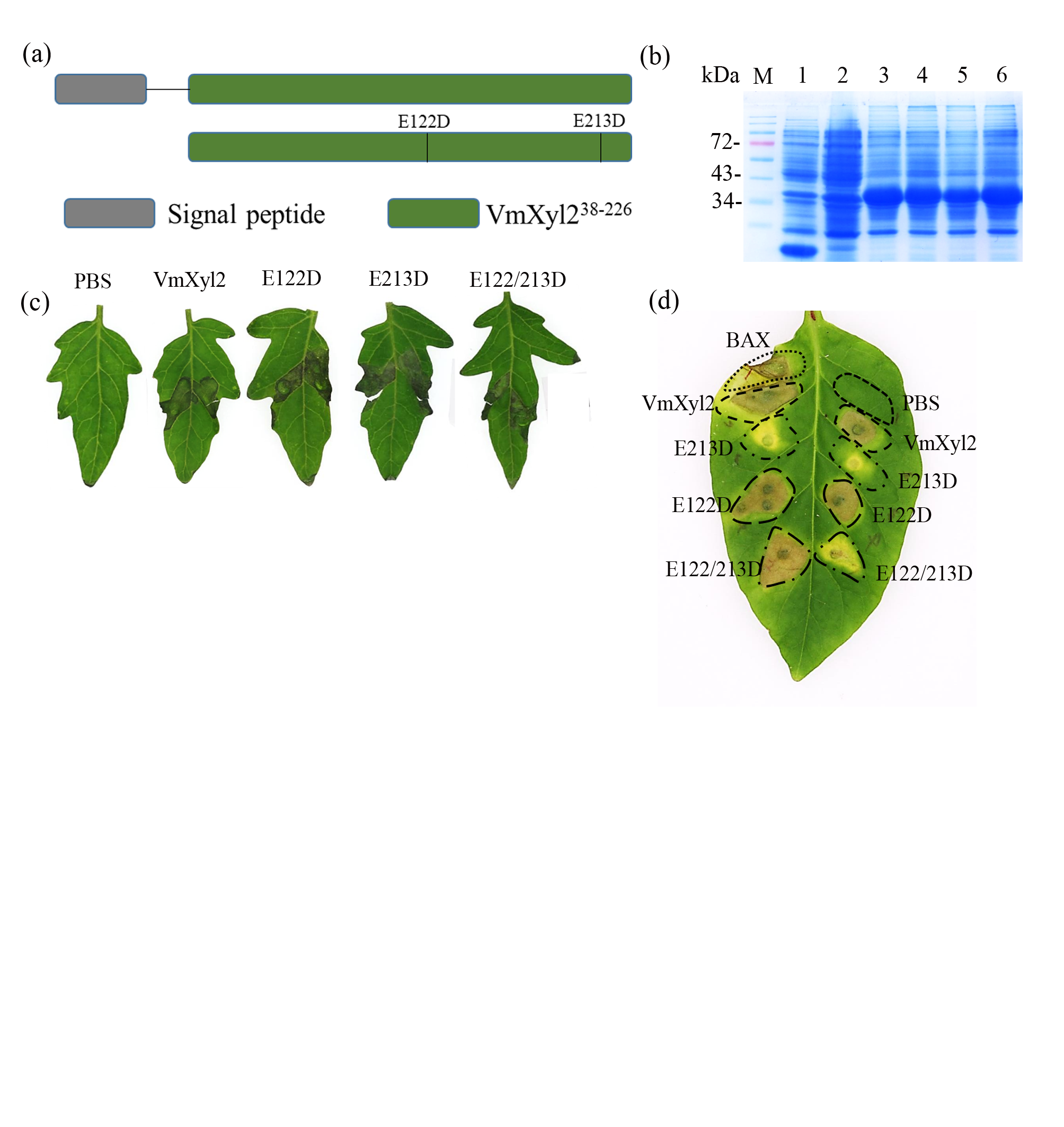

Supplement: Supplementary Figure 4 — The effect of xylanase activity on the cell death-inducing activity of VmXyl2. (A) Schematic diagram of xylanase activity site in VmXyl2. (B) Prokaryotic expression of site-directed mutant proteins. M represents protein standard, Lane 1 represents uninduced pET-32a, Lane 2 represents uninduced of pET-VmXyl2, Lane 3 represents E122D site-directed mutant protein of VmXyl2; Lane 4 represents E213D site-directed mutant protein of VmXyl2; Lane 5 represents E122D and E213D double site-directed mutant protein of VmXyl2. (C) Tomato leaves infiltration with site-directed mutant protein and wild-type protein. (D) N. tabacum cv. Samsun leaves infiltrated with site-directed mutant protein and or transiently expressing protein. [file Image_4.tif]

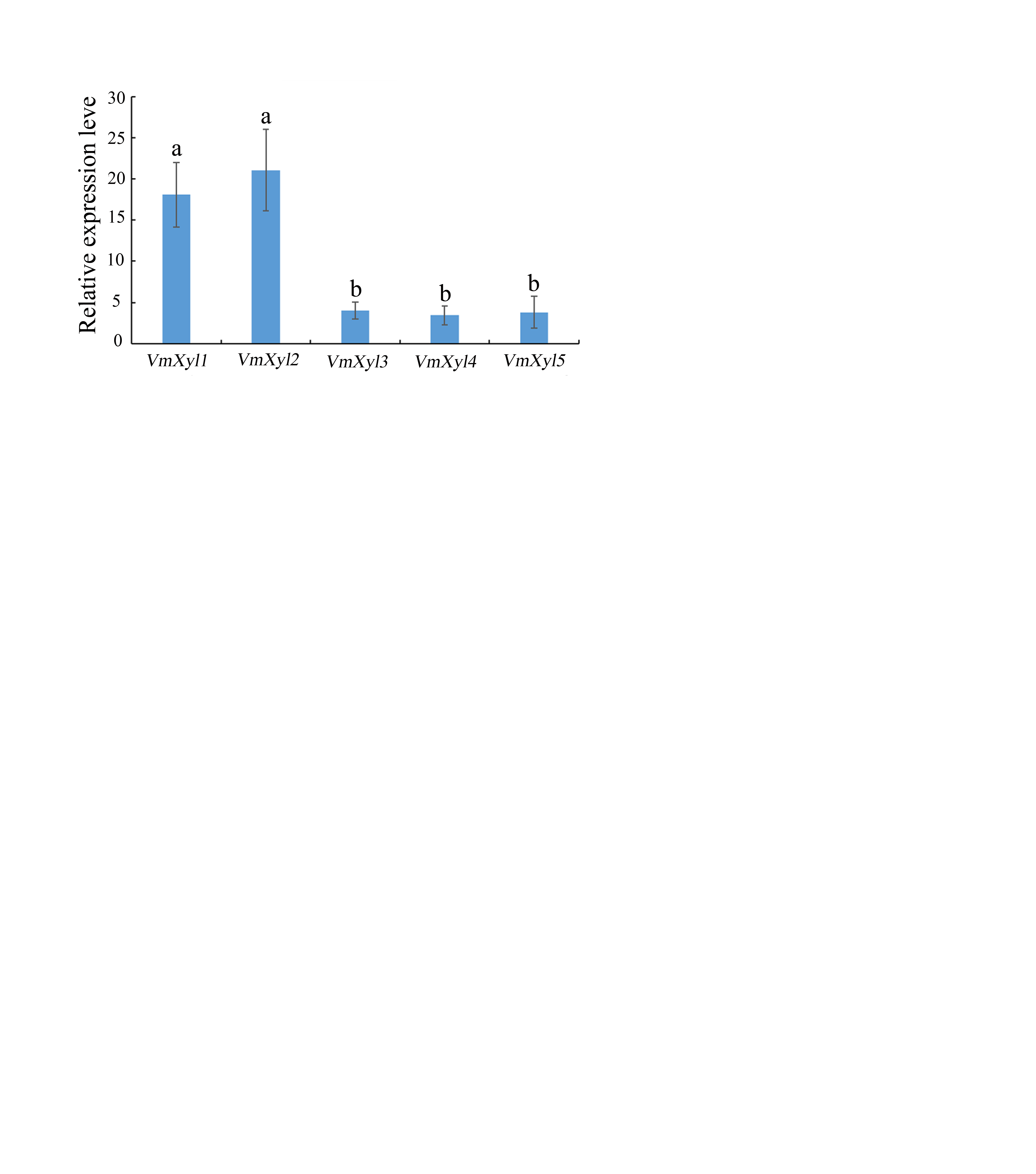

Supplement: Supplementary Figure 5 — Transcript levels of VmXyl2 at 72 h post inoculation of V. mali in apple twigs by qPCR. The transcript level of V. mali EF1-a was used as an internal control, and the transcript level of VmXyl2 in the mycelia grown on PDA was standardized to 1. The means and standard deviation of the relative expression levels were calculated from three independent biological replicates. Different letters on bars indicate a statistically significant difference (** p < 0.01). [file Image_5.tif]
