# Supplementary material for: Xylanase VmXyl2 is involved in the pathogenicity of Valsa mali by regulating xylanase activity and inducing cell necrosis
Source: Front Plant Sci. 2024 Apr 29;15:1342714. doi: 10.3389/fpls.2024.1342714 (PMC11092374; doi:10.3389/fpls.2024.1342714)
Supplement: Supplementary file 6 [file DataSheet_1.docx]

Table S1

| Primer name | Primer sequence | Description |
| --- | --- | --- |
| RTVmXyl2-F | GGCACCCAGACGTTTAACCAG | VmXyl2 fluorescent quantitative PCR amplification primer |
| RTVmXyl2-R | GCTGCTCTCATAGCCCTCGGTA |  |
| VmXyl2-Up-F | ACGACAGCATAGATACTTGG | Amplification of upstream primers of target genes |
| VmXyl2-Up-R | caaaataggcattgatgtgttgacctccTGAGTGTGTATTTGCAGTG |  |
| VmXyl2-Down-F | ctcgtccgagggcaaaggaatagagtagGGTGGCTGTTGAAATGGAAT | Amplification of downstream primers of target genes |
| VmXyl2-Down-R | GTGGATGGATGTGTGGGAAT |  |
| VmXyl2-nest-F | AGAGGAAGCGGTGTTGTCAT | Primers for amplification of upstream - HPH-downstream fusion fragments |
| VmXyl2-nest-R | TCGCTTCTACTCCCGCAACG |  |
| HPH-F | GGAGGTCAACACATCAATGCC | Primers for amplification of HPH fragments |
| HPH-R | CTACTCTATTCCTTTGCCCTCGG |  |
| VmXyl2-YH-F | gctgatatcggatccgaattcCCTACGGACGTCGCCAA | Primers for VmXyl2 prokaryotic expression |
| VmXyl2-YH-R | tggtggtgctcgagtgcggccgcTCACGTAATCGTGATA |  |
| VmXyl2-SMQF | CCCATCGATATGCTGTCCCTTCGAAACTTC | Primer of VmXyl2 attached to PVX vector |
| VmXyl2-SMQR | ATAGTTTAGCGGCCGCTCACGTAATCGTGATA |  |
| VmXyl2-QF | CCCATCGATCCTACGGACGTCGCCAA | Primers VmXyl2 without signal peptide attached to PVX vector |
| VmXyl2-QR | ATAGTTTAGCGGCCGCTCACGTAATCGTGATA |  |
| TRV2-BAK1-F | GTGAGCTCGGTACCGGATCCGTGAGGGTGGTGAGCGGGATAAT | Primers of TRV vector in *N. tabacum cv. Samsun* |
| TRV2-BAK1-R | TGAGTAAGGTTACCGAATTCGCTCATAACTGGGCAAAGGGCTT |  |
| TRV2-SOBIR1-F | GTGAGCTCGGTACCGGATCCAATCTTTATCCACCAGATCATGC |  |
| TRV2-SOBIR1-R | TGAGTAAGGTTACCGAATTCCAGAAAGTTTTCCAATGGCAG |  |
| NbSerk3-sil-EcoR1-F | ggaattcgcccttaactgggcaacgggct |  |
| NbSerk3-sil-BamH1-R | cgggatccgtgagggtggtgagcaggataa |  |
| qRT-NbBAK1-F | GAGGTGGGAGGAATGGCAAA | Primers of fluorescence quantitative detection of resistance genes |
| qRT-NbBAK1-R | TTGGCCCCGACAATTCATCT |  |
| qRT-NbSOBIR1-F | CCAGCAAGTCACAGAAGGGA |  |
| qRT-NbSOBIR1-R | CCAACACCACACCAAAGCTG |  |
| NbSerk3-qrt-F | TCCTGACGGACCATCTCCTCTTT |  |
| NbSerk3-qrt-R | GCTCATAACTGGGCAAAGGGCTT |  |
| Y/CYP-F | TTCCTCTCCATGGCTAATGC | Primers of reference genes in *N. tabacum cv. Samsun* |
| Y/CYP-R | ATCCAGATCCAACAGCCTCA |  |
| F/ACT-R | ATACGCATCCTTCTGTCCCATTCCGA | Primers of PTI pathway fluorescent quantitative PCR amplification in *N. tabacum cv. Samsun* |
| NbPti5-F | CCTCCAAGTTTGAGCTCGGATAGT |  |
| NbPti5-R | CCAAGAAATTCTCCATGCACTCTGTC |  |
| NbAcre31-F | AATTCGGCCATCGTGATCTTGGTC |  |
| NbAcre31-R | GAGAAACTGGGATTGCCTGAAGGA |  |

Table S2

Effect of VmXyl2 point mutation on xylanase activity

| Enzyme | | Xylanase activity（U/ml） | Enzyme activity ratio % |
| --- | --- | --- | --- |
| VmXyl2 | 1000 | | 100 |
| E122A | 10.09 | | 1.009 |
| E213A | 12.36 | | 1.236 |
| E122213A | 5.298 | | 0.5298 |
